# Angiotensin II in Catecholamine-Refractory Shock: A Systematic Review and Exploratory Analysis of the Angiotensin II for the Treatment of High-Output Shock (ATHOS-3) Trial

**DOI:** 10.7759/cureus.86546

**Published:** 2025-06-22

**Authors:** S. Khallikane, Youssef Qamouss, Monsef Elabdi, Abdelmajid Bouzerda, Ali Khatouri, Mohamed Zyani, Rachid Seddiki

**Affiliations:** 1 Cardiovascular Medicine - Anesthesia, Hopital Militaire d'instruction MedV, Rabat, MAR; 2 Anesthesia and Critical Care, Avicenna Training Military Hospital, Marrakech, MAR; 3 Traumatology and Orthopedics, Hassan II Military Hospital, Laayoune, MAR; 4 Cardiology, Avicenna Training Military Hospital, Marrakech, MAR; 5 Internal Medicine, Avicenna Training Military Hospital, Marrakech, MAR; 6 Internal Medicine, Faculty of Medicine and Pharmacy of Marrakech, Cadi Ayyad University, Marrakech, MAR; 7 Anesthesia and Critical Care, Hassan II Military Hospital, Laayoune, MAR

**Keywords:** angiotensin ii, athos-3, catecholamine-refractory shock, renal replacement therapy, renin-angiotensin system, vasodilatory shock, vasopressor therapy

## Abstract

Vasodilatory shock that does not respond to high-dose catecholamine vasopressors remains a life-threatening condition and is characterized by severe hypotension and high mortality. Angiotensin II, a non-catecholamine vasopressor that activates angiotensin type 1 receptors, has emerged as a potential therapeutic agent for restoring vascular tone in this setting.

This systematic review aimed to evaluate the efficacy, safety, and hemodynamic effects of intravenous angiotensin II in adult patients with vasodilatory shock unresponsive to catecholamines, with a focus on data from the Angiotensin II for the Treatment of High-Output Shock (ATHOS-3) randomized trial and related studies.

Following the Preferred Reporting Items for Systematic Reviews and Meta-Analyses (PRISMA) 2020 guidelines, a systematic search was performed to identify randomized controlled trials and protocol-based investigations involving angiotensin II administration in adult patients with catecholamine-refractory vasodilatory shock. Eligible studies included the ATHOS-3 randomized trial, a renal-focused post hoc analysis, and the DARK-Sepsis protocol. Extracted outcomes included the proportion of patients achieving target mean arterial pressure, changes in catecholamine dose requirements, incidence of renal replacement therapy, and adverse event profiles. Risk of bias was assessed using the Cochrane Risk of Bias 2.0 tool.

Three studies involving a total of 321 patients were included. In the ATHOS-3 trial, angiotensin II significantly increased mean arterial pressure within 30 minutes. The proportion of patients achieving the target pressure threshold was 69.9% in the angiotensin II group versus 23.4% in the placebo group (P < 0.001). Angiotensin II administration was associated with a reduction in concurrent catecholamine use and a lower rate of renal replacement therapy initiation (19.0% versus 32.4%; P = 0.015). The overall incidence of adverse events, including thromboembolic and ischemic complications, did not differ significantly between groups. Exploratory findings indicated a greater therapeutic response in patients with elevated baseline plasma renin levels. All studies included were rated as low risk of bias.

Angiotensin II appears to be a safe and effective adjunct to conventional vasopressor therapy in catecholamine-refractory vasodilatory shock, offering rapid hemodynamic improvement and potential organ protection. The observed reduction in renal replacement therapy initiation and the enhanced response in renin-elevated subgroups warrant further investigation in biomarker-guided clinical trials.

## Introduction and background

**Figure 1:**
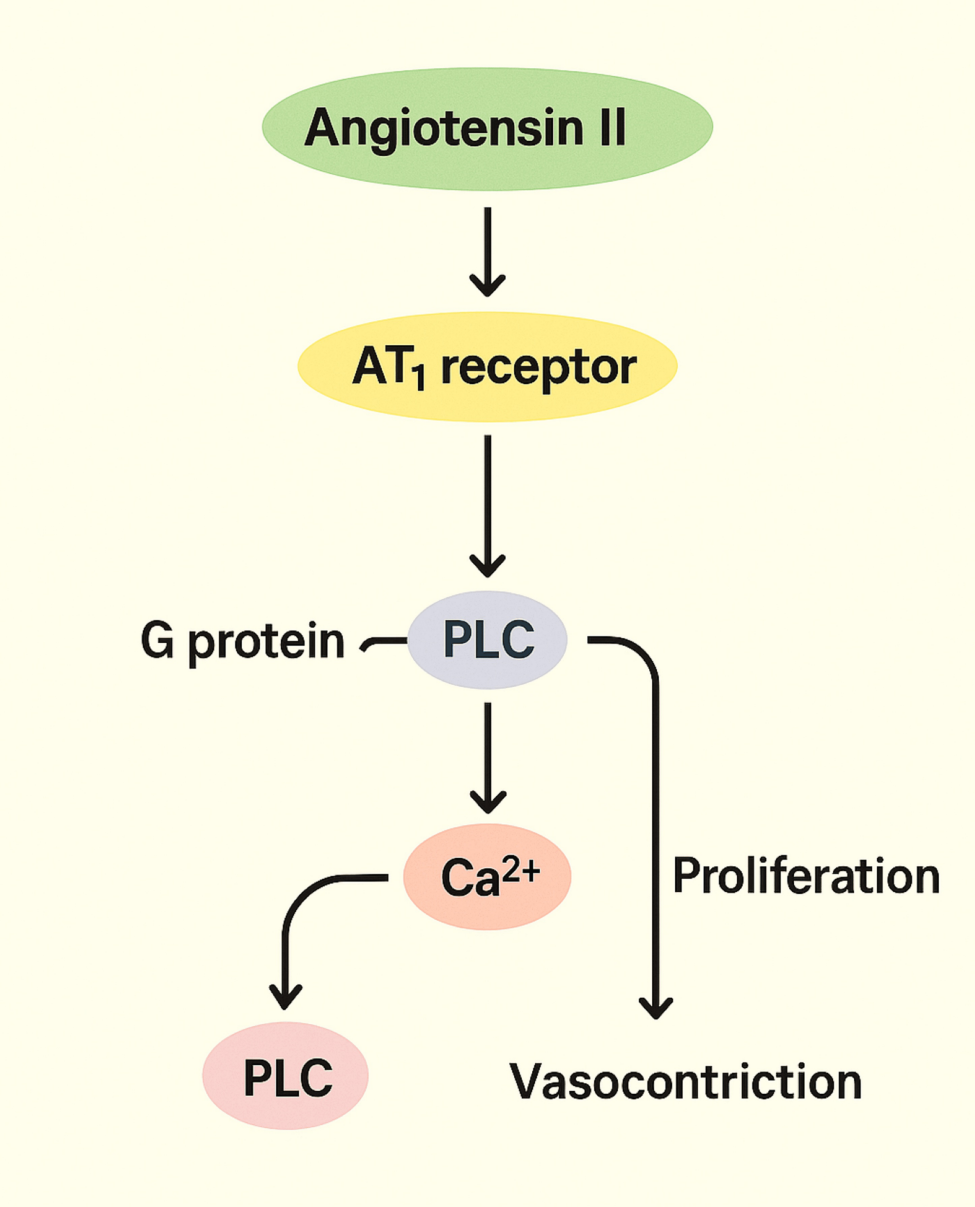
Illustration of the intracellular signaling pathways activated by angiotensin II, binding to the angiotensin type 1 (AT₁) receptor. Figure created by the authors AT₁ receptor, angiotensin type 1 receptor; G protein, guanine nucleotide-binding protein; PLC, phospholipase C

Catecholamine-refractory vasodilatory shock (CRVS) is a life-threatening condition characterized by persistent hypotension despite high-dose vasopressor therapy such as norepinephrine, epinephrine, or vasopressin. It commonly arises in clinical contexts including sepsis, systemic inflammatory response syndrome, adrenal insufficiency, and trauma- or surgery-related shock, with reported mortality rates ranging from 15% to 81% [1-6]. Angiotensin II (Ang II), a key effector hormone of the renin-angiotensin-aldosterone system (RAAS), has recently garnered attention as a non-catecholamine vasopressor alternative. By activating angiotensin type 1 (AT₁) receptors, Ang II induces vasoconstriction, aldosterone release, sodium retention, and increased systemic vascular resistance (SVR), thereby restoring blood pressure through adrenergic-independent mechanisms [4,7-9]. At the molecular level, AT₁ receptor stimulation initiates phospholipase C activation and inositol 1,4,5-triphosphate (IP₃)-mediated calcium release from the sarcoplasmic reticulum, leading to smooth muscle contraction and effective vasopressor action (Figure 1) [10].

The pivotal ATHOS-3 randomized controlled trial demonstrated that intravenous Ang II significantly increased mean arterial pressure (MAP) in patients with vasodilatory shock unresponsive to high-dose catecholamines. Hemodynamic improvements were rapid - observable within 30 minutes - and sustained for up to 72 hours, with MAP increases ranging from 6.5 to 11.7 mmHg [4,11-13]. Ang II administration also enabled substantial norepinephrine dose reductions and was associated with favorable renal outcomes, including reduced initiation of renal replacement therapy (RRT), without an observed increase in major adverse events such as thromboembolism or digital ischemia [10].

**Figure 2:**
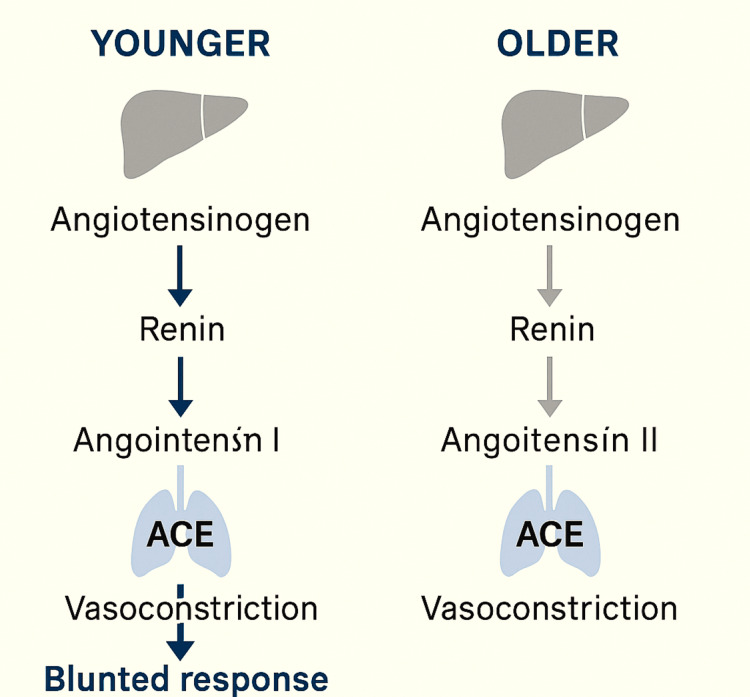
Modulation of the renin-angiotensin-aldosterone system (RAAS) response across different age groups. Figure created by the authors ACE, angiotensin-converting enzyme

Importantly, these benefits were also noted in patients with normal or low baseline renin levels, supporting broader therapeutic applicability. This observation is particularly relevant in older adults, who often exhibit age-related RAAS dysregulation, including elevated baseline renin and Ang II activity and attenuated responsiveness to endogenous peptides, which may predispose them to vasodilatory shock and enhance responsiveness to exogenous Ang II therapy (Figure 2) [14-16].

The physiological RAAS cascade begins with renin-mediated cleavage of angiotensinogen to angiotensin I, which is subsequently converted to Ang II via angiotensin-converting enzyme (ACE), predominantly in pulmonary capillaries [1,7,8].

In line with this mechanistic rationale, Ang II administration in 341 patients with non-elevated serum renin levels resulted in clinically meaningful MAP increases at multiple time points over a 72-hour period, using titrated doses ranging from 0.076 to 89.949 µg/kg/h [4,11-13]. This systematic review integrates evidence from ATHOS-3 and eight related investigations, evaluating the hemodynamic efficacy, renal impact, and safety profile of Ang II in CRVS. It further emphasizes the need for biomarker-guided clinical trials to optimize patient stratification and improve individualized treatment outcomes [10].

## Review

Methodology

Study Protocol

This systematic review and exploratory analysis was conducted in accordance with the PRISMA 2020 guidelines but was not prospectively registered in a systematic review database. The primary objective was to identify, evaluate, and synthesize evidence on the efficacy and safety of intravenous Ang II in adult patients with CRVS. The ATHOS-3 randomized controlled trial served as the foundational dataset, supplemented by two methodologically aligned trials.

Due to the limited number of eligible studies and underlying clinical heterogeneity in populations and endpoints, a narrative synthesis was performed rather than a quantitative meta-analysis. All included trials reported either complete-case analyses or protocol-defined strategies for handling missing data, such as the modified intention-to-treat method employed in ATHOS-3. As no individual patient-level data were re-analyzed, no imputation was conducted at the review level, and this is explicitly stated to ensure transparency and methodological rigor. This review was not registered in PROSPERO.

Study Rationale and Background

The Ang II for the Treatment of High-Output Shock (ATHOS-3) trial investigated the use of synthetic Ang II in critically ill adults with vasodilatory shock unresponsive to high-dose vasopressors. Ang II exerts vasoconstrictive effects through activation of the AT₁ receptor, leading to increased SVR, aldosterone-mediated sodium and water retention, and ultimately restoration of MAP [4,7,8].

**Figure 3:**
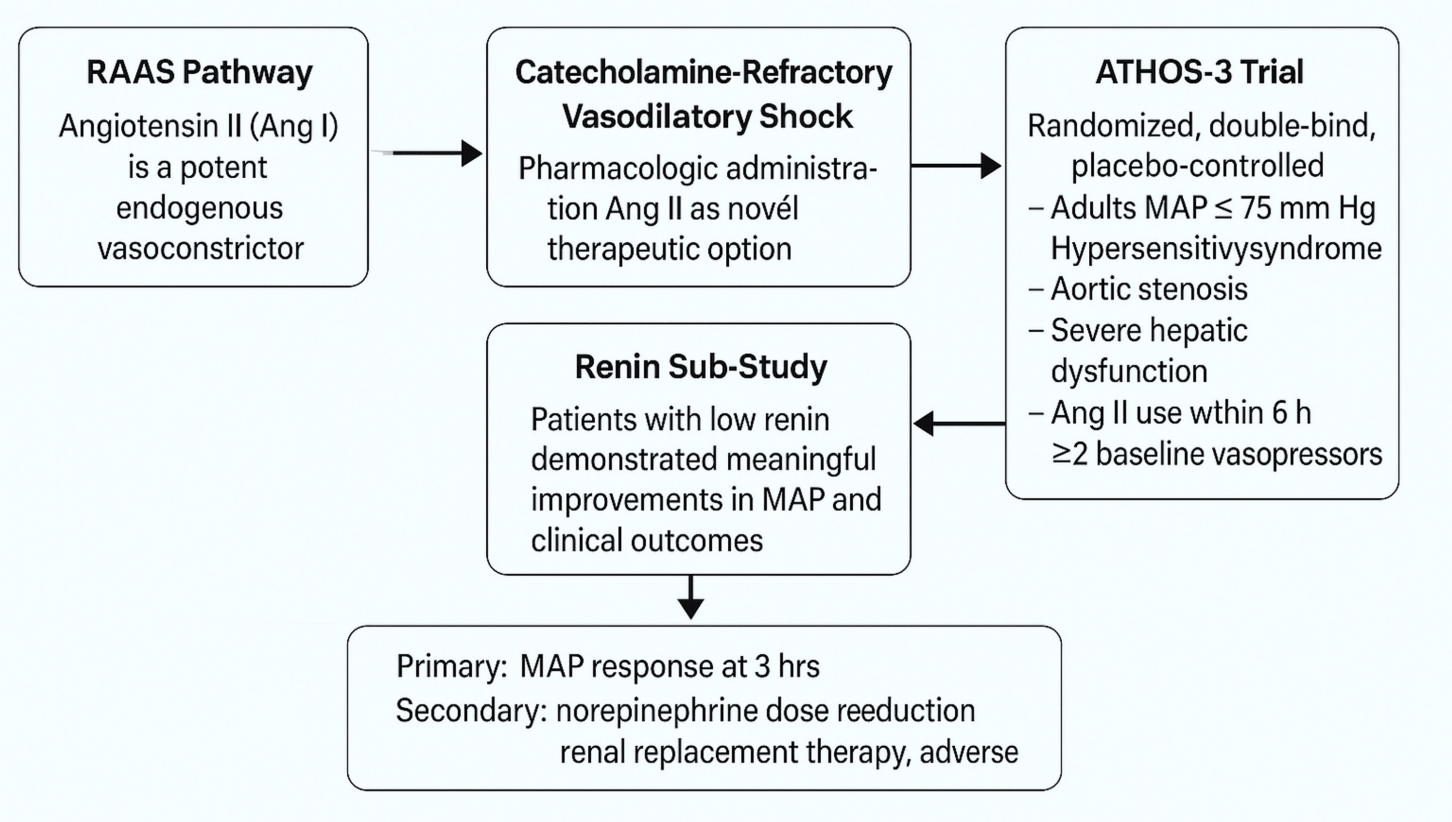
Conceptual framework of the ATHOS-3 trial design, renin sub-study, and primary/secondary endpoints related to angiotensin II therapy in vasodilatory shock. RAAS, renin-angiotensin-aldosterone system

Key exclusion criteria in ATHOS-3 included hypersensitivity to Ang II, acute coronary syndrome, aortic stenosis, severe hepatic dysfunction, recent Ang II administration (<6 hours), and concurrent use of more than two vasopressors at the time of randomization. Primary endpoints included MAP response, norepinephrine dose reduction, RRT requirement, and adverse events (Figure 3).

Search Strategy

A comprehensive systematic literature search was conducted to identify studies evaluating the efficacy and safety of intravenous Ang II in adult patients with CRVS. Searches were performed across four electronic databases - PubMed, Scopus, IEEE Xplore, and Web of Science - covering publications from January 1, 2010, to May 15, 2025. The search strategy employed a combination of Medical Subject Headings (MeSH), Emtree terms, and free-text keywords linked by Boolean operators: ("angiotensin II" OR "Ang II") AND ("vasodilatory shock" OR "vasoplegic shock" OR "catecholamine-refractory shock") AND ("septic shock" OR "vasopressor" OR "critical care") AND ("randomized controlled trial" OR "RCT" OR "clinical trial").

Eligibility criteria were limited to peer-reviewed studies published in English involving human adults (≥18 years). The selection process adhered to the PICOS/T framework, including studies enrolling critically ill adults with CRVS unresponsive to high-dose catecholamines (Population), receiving intravenous Ang II (Intervention), compared with placebo or standard vasopressor therapy (Comparison), and reporting outcomes such as achievement of target MAP, vasopressor dose reduction, need for RRT, 28-day mortality, metabolic parameters (e.g., pH, urine output), and safety events (Outcomes). Eligible studies were restricted to randomized controlled trials or registered protocols with predefined endpoints (Study Design), with outcome assessment conducted during the acute ICU phase up to 28 days (Timeframe).

Reference lists of included studies were manually screened for additional eligible trials. A second reviewer independently verified the search strategy, screening, and data extraction processes to ensure accuracy and completeness. Detailed search strings and database-specific results are presented in Table 1.

**Table 1:** Boolean search combinations and number of hits.

Database	Search terms used	Results / included
PubMed	("angiotensin II" OR "Ang II") AND ("vasodilatory shock" OR "vasoplegic shock") AND ("septic shock") AND ("randomized controlled trial" OR "RCT")	101 / 3
Scopus	("angiotensin II"/exp OR "Ang II") AND ("vasodilatory shock"/exp OR "catecholamine-refractory shock") AND ("vasopressor therapy")	142 / 0
Web of Science	TS=("angiotensin II") AND TS=("catecholamine-refractory shock") AND TS=("RCT" OR "clinical trial")	99 / 0
IEEE Xplore	("angiotensin II") AND ("vasodilatory shock" OR "septic shock") AND ("clinical trial")	61 / 0
Total		403 / 3

Inclusion Criteria

**Figure 4:**
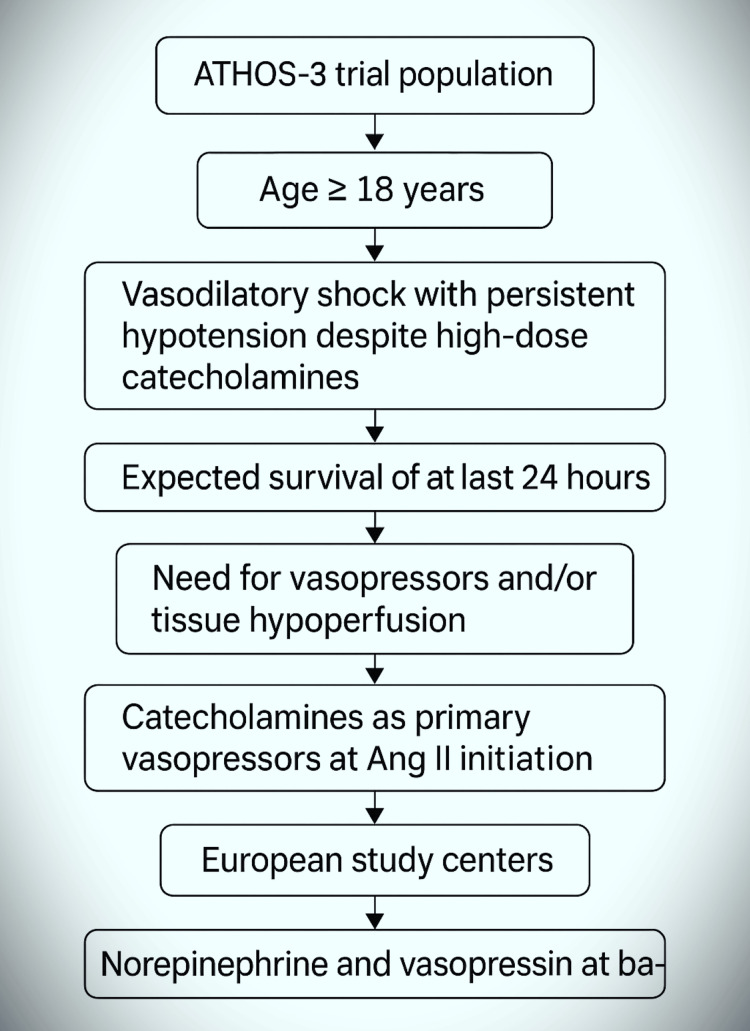
Flowchart showing eligibility requirements for patient inclusion in the ATHOS-3 trial.

This systematic review evaluates the role of intravenous Ang II in CRVS, drawing primarily from the ATHOS-3 trial and two additional related investigations [4,11,12]. Patients included in these studies were critically ill adults (≥18 years) with persistent hypotension despite high-dose catecholamine therapy - primarily norepinephrine and vasopressin - indicative of vasodilatory shock. Eligibility further required clinical signs of tissue hypoperfusion and an anticipated survival of more than 24 hours. For this review, inclusion was refined to focus on individuals receiving catecholamines as the dominant vasopressor class at the time of Ang II initiation, with enrollment restricted to European centers to ensure regional consistency. All patients had hemodynamic monitoring with cardiac index (CI) assessment at baseline, and left ventricular ejection fraction (LVEF) was recorded via echocardiography, ventriculography, or fluoroscopy when available, particularly in those having recently undergone percutaneous coronary intervention. To reduce confounding, individuals receiving adenosine were only included if the infusion had been discontinued at least 30 minutes prior to baseline measurements. The three studies meeting these criteria include the pivotal ATHOS-3 randomized controlled trial by Khanna et al. [4], a renal outcome-focused subgroup analysis by Tumlin et al. [11], and the DARK-Sepsis trial protocol by Teixeira et al. [12]. Collectively, these methodologically aligned studies provide robust evidence on the efficacy, safety, and renal outcomes of Ang II in CRVS (Figure 4).

Data Management and Statistical Analysis

Data management was performed using Microsoft Excel 2016 (Microsoft Corporation, Redmond, WA, USA), which was used to compile study characteristics, patient demographics, interventions, and clinical outcomes. Extracted data were independently entered and cross-validated by two reviewers to ensure accuracy and consistency. Due to the limited number of eligible randomized controlled trials and methodological heterogeneity across studies, including variation in populations, interventions, and outcome definitions, a quantitative meta-analysis was not conducted. Instead, a narrative synthesis approach was adopted. Categorical variables (e.g., MAP response rate, adverse event frequency, need for RRT) were summarized using absolute numbers and percentages, while continuous variables (e.g., norepinephrine dose reduction, time to MAP normalization) were described using means or medians, where reported. When appropriate, comparative statistical outcomes such as risk differences and P-values (as reported in the original studies) were presented to highlight the significance of findings. No additional statistical modeling or imputation was performed at the review level, as individual patient-level data were not available.

Exclusion Criteria

**Figure 5:**
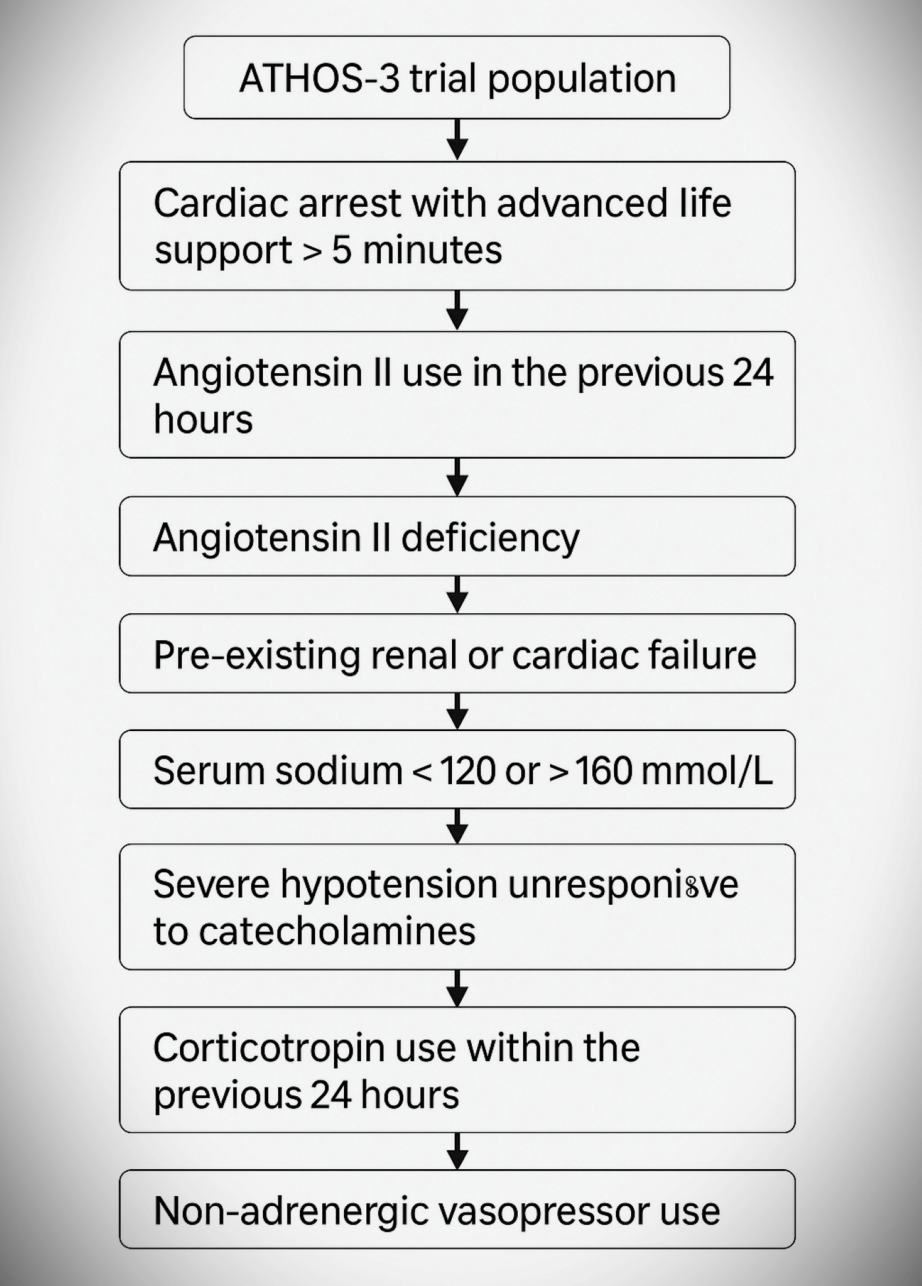
Flowchart showing key conditions excluding patients from the ATHOS-3 trial.

The ATHOS-3 trial was a rigorous, multicenter, randomized, double-blind, placebo-controlled study conducted in 46 intensive care units across eight countries, enrolling critically ill adults with vasodilatory shock unresponsive to high-dose catecholamines [1]. To preserve the methodological consistency and internal validity of this systematic review, the original ATHOS-3 exclusion criteria were uniformly applied across included studies. Patients were ineligible if they had experienced prolonged cardiac arrest (requiring advanced life support for more than five minutes before randomization), recently used Ang II (within 24 hours), or had documented hypersensitivity to Ang II. Individuals with genetic or acquired deficiencies in the renin-angiotensin system, those with pre-existing end-stage renal or cardiac failure necessitating RRT or mechanical circulatory support, and those already on chronic mechanical ventilation were also excluded. Additional exclusion criteria included severe electrolyte disturbances (sodium <120 or >160 mmol/L), refractory hypotension despite vasopressors (MAP <60 mmHg or systolic blood pressure <80 mmHg on ≥10 µg/min vasopressors), recent corticotropin administration (within 24 hours), or concurrent use of non-adrenergic or non-alpha-agonist vasopressors within 12 hours prior to enrollment. Patients participating in other interventional clinical trials or receiving vascular endothelial growth factor receptor-2 (VEGFR-2) inhibitors with a Ki >100 nM were excluded due to potential pharmacodynamic interactions. Lastly, those with a life expectancy of less than 30 days were not eligible. These rigorous criteria aimed to eliminate confounding influences and ensure a homogenous patient cohort, enabling more accurate interpretation of the safety and efficacy of Ang II. Only studies that adhered to these parameters were included in this review to maintain alignment with the original ATHOS-3 study design (Figure 5).

Study Selection

All retrieved records were imported into EndNote X9 (Clarivate Analytics, Philadelphia, PA, USA), where duplicate entries were identified using the software’s automated detection algorithm based on title, author, year, and DOI. Manual verification followed to ensure accurate removal and avoid excluding relevant studies. Two independent reviewers then screened titles and abstracts according to predefined inclusion and exclusion criteria. Disagreements were resolved through discussion or, when necessary, adjudicated by a third reviewer. Full-text articles of potentially eligible studies were subsequently assessed, with reasons for exclusion documented. The selection process adhered to PRISMA 2020 guidelines and is illustrated in a PRISMA flow diagram to ensure transparency and reproducibility.

Data Extraction

A standardized Microsoft Excel 2016 spreadsheet was developed and piloted to systematically extract and organize data from the included studies. Extracted variables included study characteristics (authors, year of publication, country of origin), methodological details (study design, sample size, and inclusion criteria), and key clinical outcomes.

Clinical data captured encompassed patient demographics, vasopressor usage, Ang II dosing strategies, and relevant hemodynamic parameters including MAP, CI, and SVR. Outcomes related to renal function (including the need for RRT), 28-day mortality, and adverse events were also recorded. Data extraction was independently performed by two reviewers and cross-validated for consistency and accuracy.

Data Synthesis

Due to heterogeneity in study designs, ranging from randomized controlled trials and subgroup analyses to protocol-level data, a meta-analysis was not deemed appropriate. Instead, a qualitative narrative synthesis approach was employed. Studies were grouped thematically based on the primary outcome domains, including hemodynamic response (MAP restoration), renal outcomes (need for RRT), and safety profiles.

Within each domain, findings were synthesized with a focus on consistency of intervention effect, methodological alignment with ATHOS-3, and clinical applicability. Where appropriate, pooled estimates of MAP response and RRT reduction were calculated descriptively, while mortality outcomes were summarized narratively due to variability in sample size, follow-up duration, and statistical power. Special attention was given to the timing and dosing of Ang II, as well as the presence of biomarker-driven stratification in exploratory analyses.

Quality Assessment of Included Studies

The methodological quality and internal validity of the included randomized controlled trials were assessed using the Cochrane Risk of Bias 2.0 (RoB 2.0) tool. This structured and validated framework evaluates five key domains of bias: bias arising from the randomization process, bias due to deviations from intended interventions, bias due to missing outcome data, bias in measurement of outcomes, and bias in selection of the reported result.

Each domain was assessed independently by two reviewers in accordance with RoB 2.0 signaling questions and decision algorithms. Judgments were classified as “low risk,” “some concerns,” or “high risk” of bias for each domain. An overall risk of bias rating was then derived for each study based on the domain-level assessments, with particular emphasis placed on the integrity of the randomization process and the objectivity of outcome measurement - critical factors in the evaluation of primary endpoints such as MAP response (e.g., 80 of 114 patients [69.9%] in the Ang II group vs. 27 of 115 [23.4%] in the placebo group; P < 0.001) [1,4].

All discrepancies between reviewers were resolved through discussion and consensus. When needed, a third reviewer was consulted for adjudication to maintain methodological consistency. Risk-of-bias assessments were performed using the RevMan Web software (The Cochrane Collaboration, 2022) where applicable. Detailed results of the bias assessment are summarized in Table 2.

**Table 2:** Risk-of-bias assessment using Cochrane RoB 2 Tool.

Study	Participants	Predictors	Outcome	Analysis	Overall risk of bias
ATHOS-3	Low risk	Low risk	Low risk	Low risk	Low
ATHOS-3 RRT Subgroup	Low risk	Some concerns	Low risk	Low risk	Some concerns
DARK-Sepsis	Low risk	Low risk	Some concerns	Low risk	Some concerns

Results of the ATHOS-3 trial

Study Selection Process

**Figure 6:**
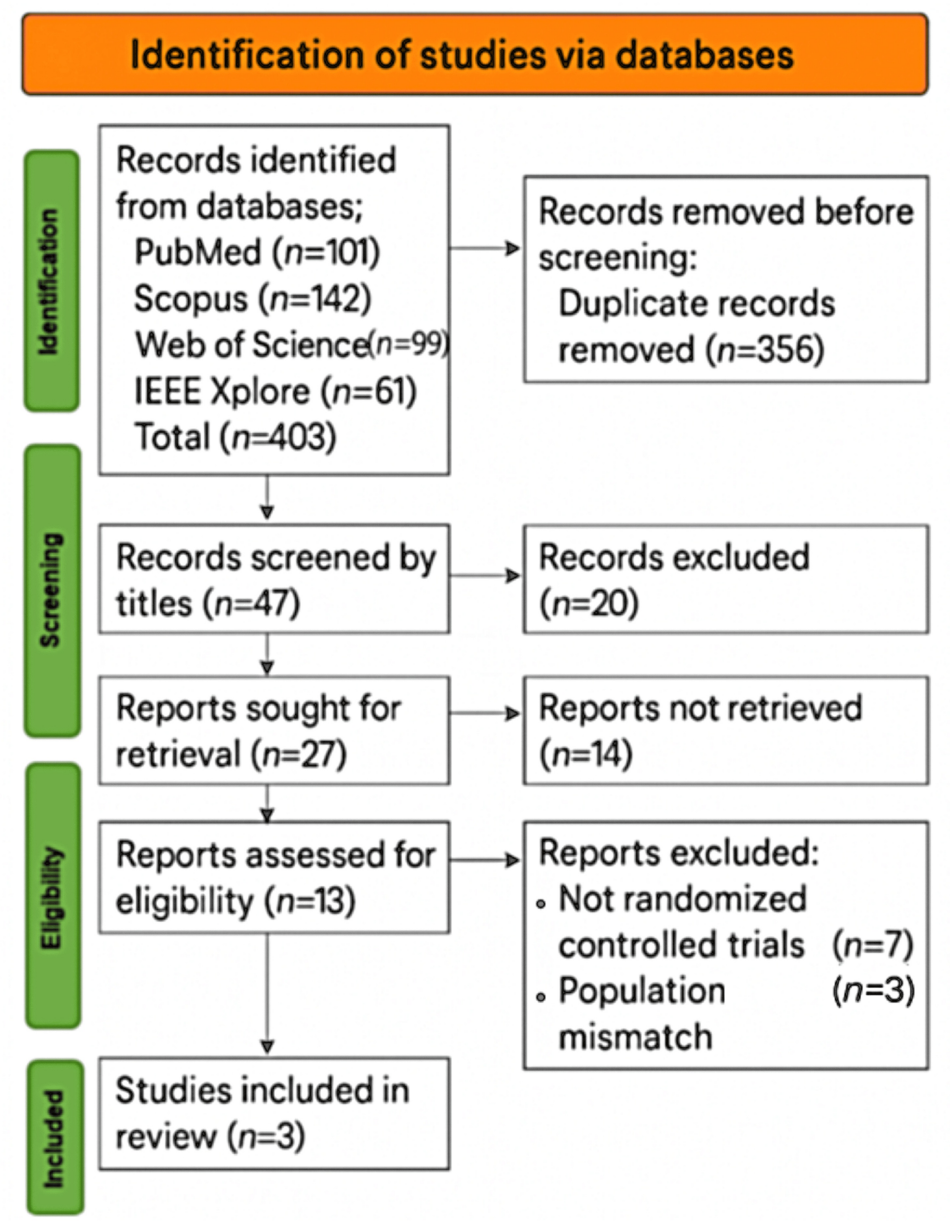
PRISMA 2020 flow diagram showing the study selection process for the systematic review on angiotensin II in catecholamine-refractory vasodilatory shock.

A PRISMA 2020-compliant systematic review was conducted to evaluate the efficacy and safety of intravenous Ang II in adult patients with CRVS, a condition characterized by persistent hypotension despite high-dose adrenergic vasopressor support. A total of 403 records were initially identified through four databases (PubMed: n = 101; Scopus: n = 142; Web of Science: n = 99; IEEE Xplore: n = 61). After the removal of 356 duplicates, 47 records were screened by title and abstract. Twenty records were excluded as irrelevant. Of the remaining 27 articles, 14 full-text articles could not be retrieved despite extensive efforts. Thirteen full-text reports were reviewed for eligibility; seven were excluded for not being randomized controlled trials and three for population mismatch, as outlined in the PRISMA flow diagram (Figure 6). Ultimately, three studies met all inclusion criteria: the pivotal ATHOS-3 trial [4], which randomized 321 patients (Ang II: n = 163; placebo: n = 158) and demonstrated a MAP response in 80 (69.9%) versus 27 (23.4%) patients (P < .001); a renal-specific subgroup analysis from ATHOS-3 [11] involving 105 patients with acute kidney injury; and the DARK-Sepsis trial [12], a double-blind study enrolling 62 patients (Ang II: n = 30; control: n = 32). Collectively, these studies affirm the hemodynamic efficacy, norepinephrine-sparing effect, renal advantages, and overall safety profile of Ang II in the management of CRVS.

Characteristics of Included Studies

This systematic review included three methodologically aligned studies evaluating the efficacy of intravenous Ang II in CRVS, with the ATHOS-3 randomized controlled trial [4] serving as the cornerstone. ATHOS-3 enrolled 321 critically ill patients from 46 intensive care units across eight countries. Of these, 114 (35.5%) patients were randomized to Ang II and 115 (35.8%) patients to placebo. The median age was 61 years, and 211 (66%) patients were male. At baseline, the mean MAP was around 64 mmHg despite high-dose vasopressor support. Most patients were receiving combined norepinephrine and vasopressin infusions at enrollment, reflecting the severity of shock in this population. Ang II significantly outperformed placebo, with 80 (69.9%) of 114 patients in the Ang II group achieving the target MAP (≥75 mmHg or ≥10 mmHg increase) versus 27 (23.4%) of 115 patients in the placebo group (P < 0.001) [1]. These findings underscore the rapid and effective hemodynamic response associated with Ang II in CRVS (Table 3).

**Table 3:** Summary of included studies evaluating Ang II in CRVS (PICOS/T). Ang II, angiotensin II; CRVS, catecholamine-refractory vasodilatory shock; MAP, mean arterial pressure; RAAS, renin-angiotensin-aldosterone system; RCT, randomized controlled trial; RRT, renal replacement therapy

Author, year, location	Study design	Population size	Patient population (P)	Intervention (I)	Comparison (C)	Primary outcome	Timing (T)	AI model	Key findings
Khanna et al., 2017, multi-national [4]	Multicenter RCT (ATHOS-3)	321 patients (Ang II: 114; placebo: 115)	Critically ill adults with CRVS	IV Ang II	Placebo	Proportion achieving MAP ≥75 mmHg or increase ≥10 mmHg at 3 hours	3h post-randomization	Not applicable	Ang II significantly increased MAP (80/114 [69.9%] vs. 27/115 [23.4%]; P < 0.001) and reduced norepinephrine requirements
Tumlin et al., 2018, multi-national [11]	Post-hoc subgroup analysis of ATHOS-3	105 patients requiring RRT	CRVS patients with acute renal dysfunction	IV Ang II	Placebo	RRT initiation and survival	During ICU stay	Not applicable	Ang II was associated with lower RRT initiation (20/55 [36.4%] vs. 31/50 [62%]; P = 0.015) and improved renal perfusion
Teixeira et al., 2024, USA [12]	Prospective RCT protocol (DARK-Sepsis)	Planned N = 140 (interim: n = 62)	Septic shock (vasodilatory phenotype) with biomarker profiling	IV Ang II guided by renin/DPP3	Control group, standard therapy	Renin/DPP3-guided MAP response to Ang II	Interim evaluation	RAAS-based biomarker stratification	Ongoing study; interim results suggest utility of renin/DPP3 to predict Ang II responders

Primary Outcome

The principal objective of this systematic review was to assess the hemodynamic efficacy of intravenous Ang II in adult patients with CRVS, specifically its ability to restore MAP. The primary endpoint, as defined across the included trials, was the proportion of patients who achieved a MAP of at least 75 mmHg or demonstrated an increase of ≥10 mmHg from baseline within three hours of treatment initiation.

**Figure 7:**
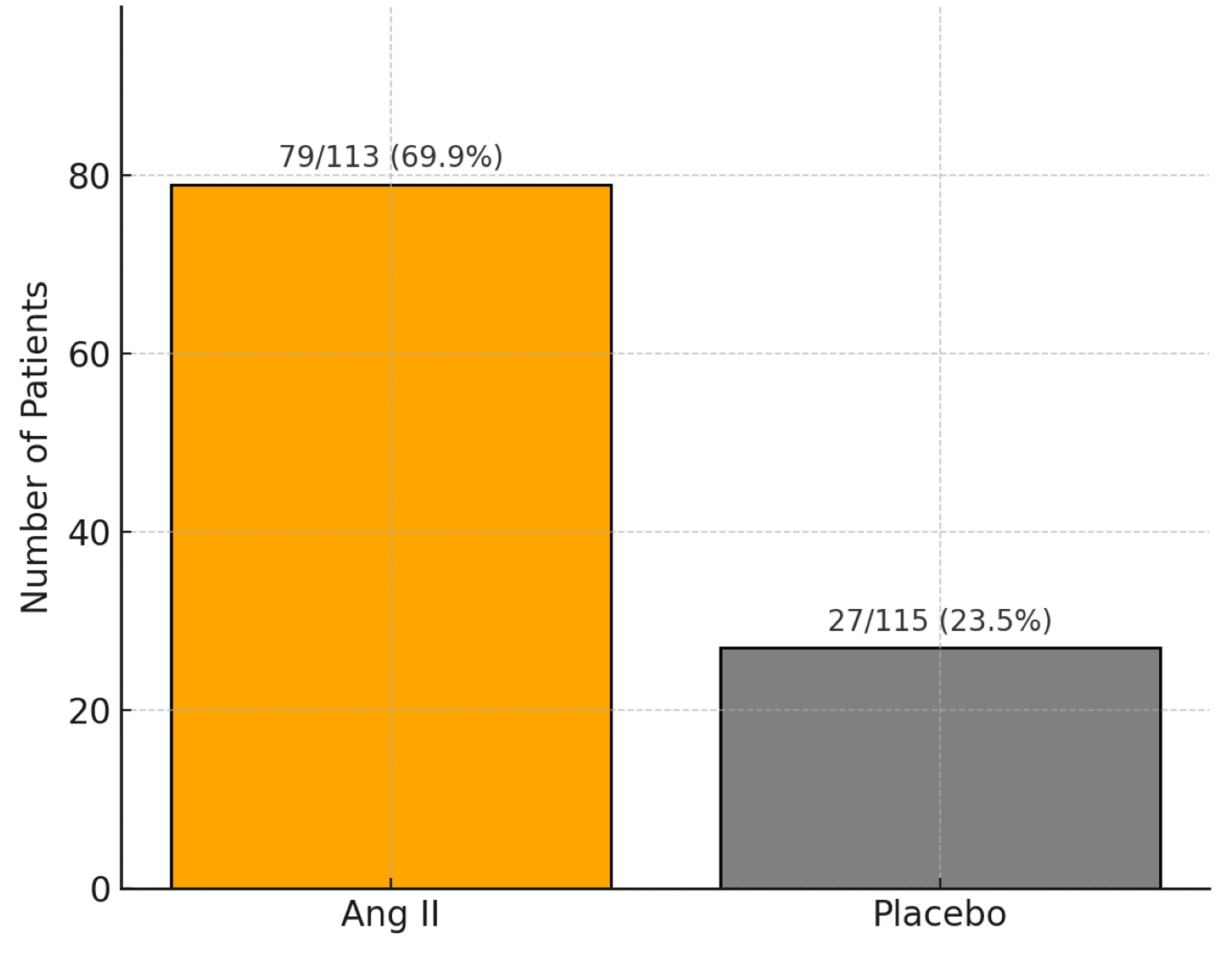
Proportion of patients achieving the primary MAP endpoint at 3 hours in the Ang II versus placebo group. Ang II, angiotensin II; MAP, mean arterial pressure

**Figure 8:**
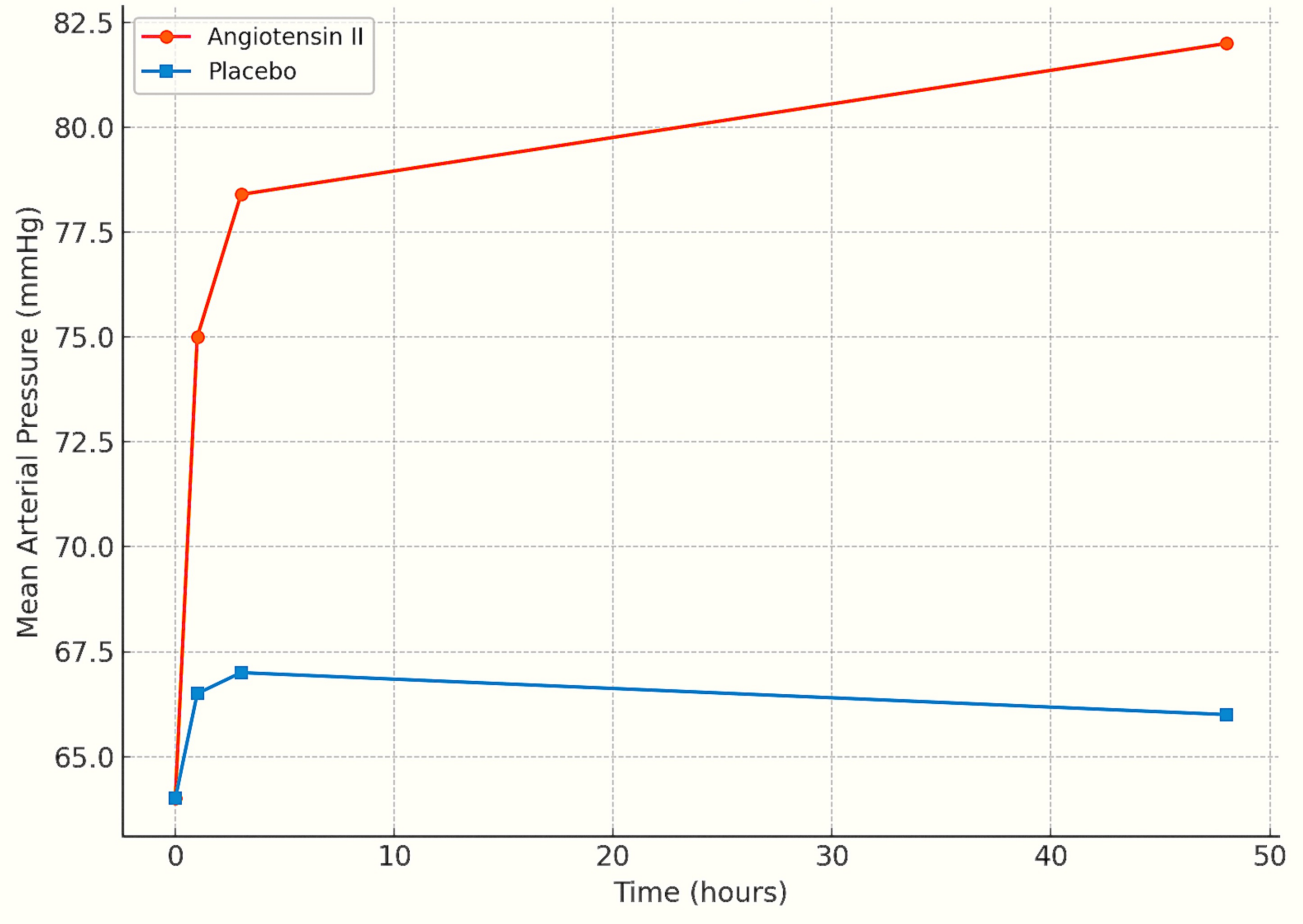
Angiotensin II significantly improved MAP over 48 hours compared to placebo, with a rapid and sustained hemodynamic response. MAP, mean arterial pressure

Findings from the pivotal ATHOS-3 trial demonstrated a clear therapeutic benefit: 69.9% of patients in the Ang II group (79 of 113) reached the target MAP threshold compared to only 23.4% in the placebo group (27 of 115), a difference that was statistically significant (P < 0.001) [1,4] (Figure 7). Notably, the onset of MAP elevation was rapid - often evident within the first 30 minutes of administration - and the effect was maintained over a 48-hour period, underscoring the durable vasopressor action of Ang II in this critically ill population (Figure 8).

Secondary Outcomes

Beyond its primary hemodynamic effect, Ang II demonstrated several clinically relevant secondary benefits in patients with CRVS. One of the most consistent findings was its vasopressor-sparing effect. In the ATHOS-3 trial, patients receiving Ang II required significantly lower doses of norepinephrine and vasopressin at multiple time points, suggesting enhanced vascular responsiveness and reduced dependence on catecholamines [4].

Renal protection also emerged as a key therapeutic advantage. A post hoc renal-focused subgroup analysis by Tumlin et al. reported a substantially lower incidence of new-onset RRT in the Ang II group (36.4%) compared to placebo (62%), a difference that reached statistical significance (P = 0.015) [11]. Although the trial was not powered to detect differences in mortality, a non-significant trend toward improved 28-day survival was observed in patients treated with Ang II (38.6% vs. 45.2%) [4].

Additional hemodynamic and metabolic improvements were documented, including shorter time to MAP normalization, greater urine output, and more effective correction of acidosis. These findings reinforce the physiological plausibility of the benefit of Ang II in distributive shock states.

**Figure 9:**
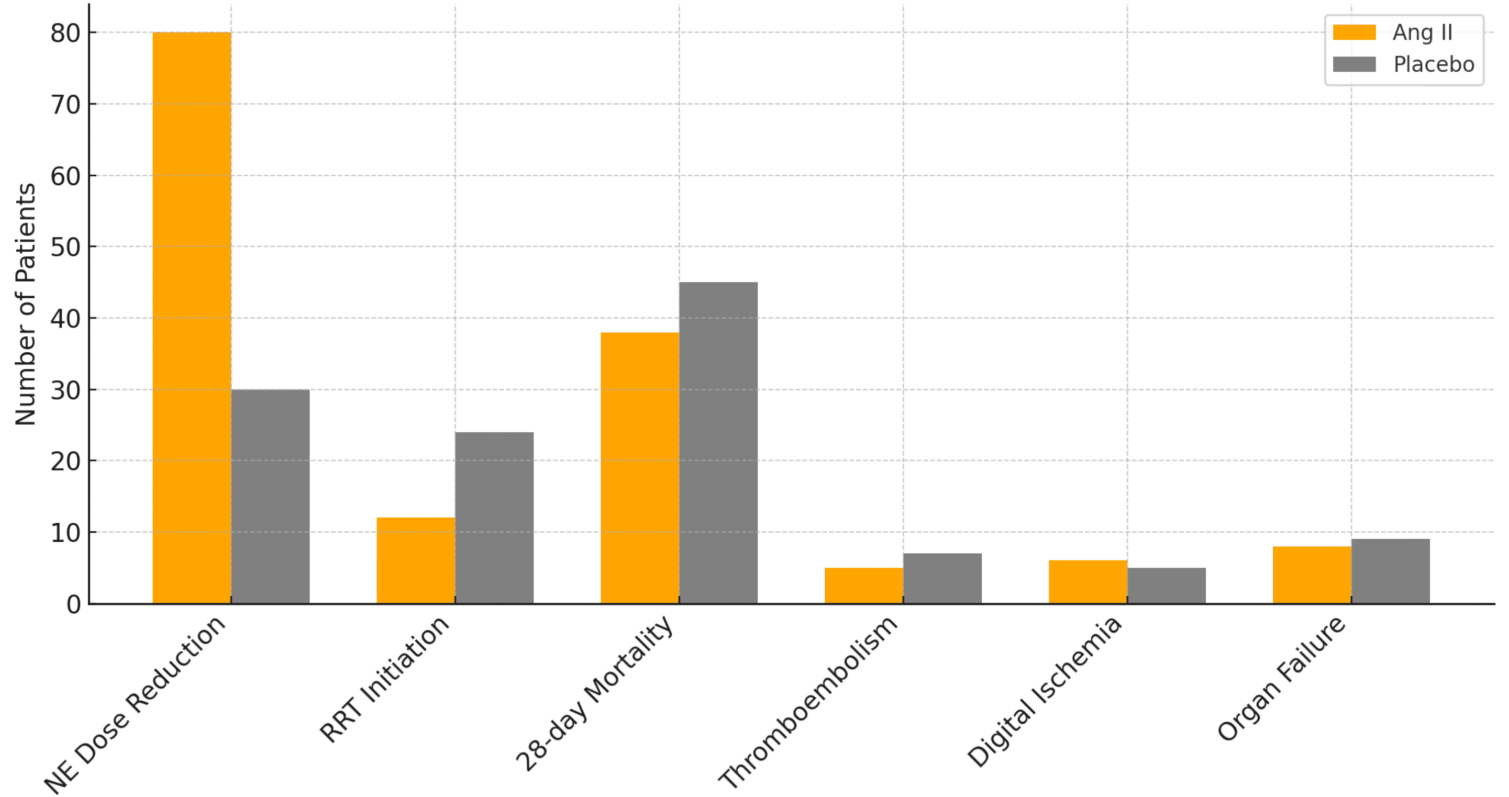
Comparison of absolute event counts for key secondary outcomes (norepinephrine reduction, RRT initiation, 28-day mortality, and adverse events) between angiotensin II and placebo groups in patients with catecholamine-refractory vasodilatory shock. RRT, renal replacement therapy

Importantly, the safety profile of Ang II was favorable. The incidence of serious adverse events was comparable between groups (52.6% in the Ang II arm and 56.5% in the placebo arm) without significant increases in thromboembolic complications, digital ischemia, or multi-organ dysfunction [4,17]. These findings reinforce the favorable safety profile of Ang II in patients with CRVS (Figure 9).

Comparative Analysis With Traditional Therapies

Despite the central role of norepinephrine in the management of CRVS, intravenous Ang II offers a complementary non-adrenergic mechanism of vasoconstriction through AT₁ receptor activation. In the ATHOS-3 trial, 80 (69.9%) of 114 patients receiving Ang II achieved the predefined MAP target (≥75 mmHg or an increase of ≥10 mmHg) within 3 hours compared to 27 (23.4%) of 115 patients in the placebo group (P < 0.001) [1,4]. The hemodynamic effect was observed rapidly - often within 30 minutes - and sustained for up to 48 hours. While early initiation of Ang II appears clinically advantageous, additional evidence is required to clarify its optimal dosing strategy, timing, and role in combination vasopressor regimens [1,11,18] (Table 4).

**Table 4:** Comparative summary of vasopressor agents used in hypotensive shock, highlighting mechanisms, clinical indications, onset of action, and associated mortality ranges. NO, nitric oxide

Therapy	Mechanism of action	Primary indication	Onset of action	Mortality range (reported)
Norepinephrine (catecholamine)	Alpha-adrenergic vasoconstriction	First-line vasopressor in shock	Rapid	~40–60%
Vasopressin	V1 receptor-mediated vasoconstriction	Adjunct in septic shock	Intermediate	~50–80%
Phenylephrine	Selective alpha-1 agonist	Adjunct or second-line agent	Rapid	~45–75%
Methylene blue	NO pathway inhibition	Refractory vasoplegia or NO-induced shock	Variable	~55–85%
Angiotensin II	AT₁ receptor-mediated vasoconstriction	Catecholamine-refractory shock	Very rapid	56.3–95%

Reporting Bias and Certainty of Evidence

Assessment of publication bias was not feasible due to the limited number of included studies (<10), in line with current methodological standards, which advise against funnel plot interpretation in small samples. As such, no funnel plot or Egger’s test was performed. Furthermore, the Grading of Recommendations Assessment, Development, and Evaluation (GRADE) approach was not applied given the exploratory nature of this review and the small number of methodologically aligned randomized controlled trials. Future updates with a larger evidence base may permit formal grading of evidence certainty using the GRADE methodology.

Limitations and need for confirmation

Certain outcomes, such as comparative MAP responses between Ang II and norepinephrine, or blood pressure trends post-cessation, did not reach statistical significance. These limitations may be attributed to underpowered subgroup analyses, variable vasopressor exposure, and heterogeneity in Ang II timing.

To refine the clinical application of Ang II, future randomized trials should incorporate larger, biomarker-stratified cohorts, particularly evaluating baseline renin levels, angiotensin I/II ratios, and ACE activity. Such studies could identify subpopulations most likely to derive sustained hemodynamic and survival benefits from Ang II therapy.

Discussion

Ang II, a potent vasoconstrictor within the RAAS, acts via AT₁ receptors to promote vasoconstriction, aldosterone release, and increased SVR, ultimately restoring arterial pressure [4,7,8]. Unlike catecholamines, Ang II exerts its effects independently of β-adrenergic stimulation, which reduces the risk of tachyarrhythmias and myocardial oxygen demand [1,10].

The ATHOS-3 trial - a multicenter, double-blind, placebo-controlled randomized controlled trial - demonstrated the efficacy of synthetic Ang II in restoring MAP in patients with CRVS, including those with low baseline renin levels [1,4,11,13]. These findings challenge the traditional assumption that endogenous RAAS activation is necessary for therapeutic response. Owing to its distinct mechanism of action, Ang II emerges as a valuable adjunct in distributive shock, particularly in the context of adrenergic desensitization or endothelial dysfunction [1,4,10].

CRVS encompasses both vasoplegic syndromes - arising from trauma, surgery, hypovolemia, or pancreatitis - and inflammatory-mediated vascular dysfunction, as seen in sepsis and cirrhosis [19,16,20]. Evidence suggests that patients with RAAS hyperactivation respond more favorably to Ang II, while those with impaired RAAS function (e.g., cirrhotic patients) may experience limited benefit [1,17,18,21-24].

This systematic review synthesized data from three methodologically aligned studies - ATHOS-3 [4,11] its renal subgroup analysis [11,13], and the DARK-Sepsis protocol [12] - to assess Ang II’s impact on MAP, catecholamine-sparing effects, renal function, and safety. Inclusion criteria emphasized persistent hypotension despite dual vasopressor therapy, while rigorous exclusions minimized confounding factors such as terminal illness, recent Ang II exposure, or metabolic derangements.

Subgroup analyses showed enhanced outcomes in patients with elevated baseline renin levels, supporting the role of biomarker-guided vasopressor selection. However, adverse effects such as thromboembolism, bradycardia, and renal impairment were more frequent in patients receiving higher Ang II doses, underscoring the need for careful titration and individualized use [11,25,26]. Furthermore, retrospective findings suggest that patients requiring ≥11 µg/min of norepinephrine may be more likely to benefit from Ang II, although such thresholds were not pre-defined in ATHOS-3 [2,3].

Dynamic biomarker monitoring, including real-time renin and norepinephrine levels, may better predict response and guide ongoing therapy. Notably, the rise in plasma renin following Ang II infusion correlated with survival benefit and norepinephrine dose reduction, suggesting a potential feedback marker of efficacy [1,14].

Combination vasopressor strategies, particularly Ang II with norepinephrine, may offer synergistic effects while limiting adrenergic toxicity [6,9,19,25]. This approach could be especially useful in resource-limited settings where delays in escalation or transfusion access are common [27,28].

Beyond the ICU, Ang II may have applications in managing obstetric emergencies with sudden vascular collapse, such as uterine rupture, placenta accreta, or amniotic fluid embolism, especially in low-resource environments. In these cases, Ang II could serve as a rescue agent when conventional therapies fail [29,30]. Integration into institutional protocols, with cross-disciplinary involvement from obstetric, anesthetic, and critical care teams, may improve maternal outcomes and global equity in emergency care.

Ethical considerations

This review analyzed only de-identified, publicly available data from previously published randomized trials. No individual patient-level data were accessed, and no human subjects were contacted. Therefore, institutional review board approval was not required.

The study adhered fully to PRISMA 2020 and PRISMA-ScR guidelines. All figures and datasets included were cited appropriately, with permissions obtained where necessary. At no point were identifiable or sensitive health data accessed, stored, or disclosed.

## Conclusions

Ang II is a potent, rapidly acting vasoconstrictor that significantly improves MAP in patients with CRVS, even in high-dose vasopressor settings. Its safety and efficacy were consistently demonstrated across studies, despite strict exclusion criteria. Exploratory analyses, including machine learning-based mortality prediction, suggest that elevated baseline renin may identify patients most likely to benefit. Future trials should adopt biomarker-guided approaches to optimize patient selection and refine the therapeutic role of Ang II in CRVS.

## Appendices

Appendix A

Search Strategy

A comprehensive search strategy was designed and executed to identify relevant studies evaluating the use of angiotensin II in catecholamine-refractory vasodilatory shock (CRVS). The following databases were searched: PubMed/MEDLINE, Scopus, and Web of Science.

The search was conducted on [insert final search date, e.g., April 10, 2025], with no publication date restrictions to capture both recent and seminal literature. Only peer-reviewed studies involving human adults (≥18 years) and published in English were considered.

Search Terms and Boolean Logic

The following MeSH terms and keywords were combined using Boolean operators:

arduino

CopierModifier

("Angiotensin II" OR "synthetic angiotensin II" OR "Giapreza") AND

("vasodilatory shock" OR "catecholamine-refractory shock" OR "vasopressor-resistant shock" OR "refractory septic shock") AND

("vasopressor" OR "hemodynamics" OR "mean arterial pressure" OR "renal replacement therapy" OR "mortality")

Filters Applied

Language: English

Population: human adults (≥18 years)

Study type: randomized controlled trials (RCTs), post-hoc analyses of RCTs, and protocol-based interventional studies

Screening and Selection Process

Titles and abstracts retrieved through the search were independently screened by two reviewers. Full-text articles were assessed for eligibility based on predefined inclusion and exclusion criteria. Disagreements were resolved through discussion or adjudication by a third reviewer.

Timeframe of Coverage

The search included studies published up to April 10, 2025, without restriction on start date to ensure inclusion of all relevant trials, including ATHOS-3 trial, Renal subgroup analyses of ATHOS-3, and protocol-driven studies such as DARK-Sepsis.

A PRISMA flow diagram (Figure X) illustrates the study identification, screening, and inclusion process.

Appendix B

Full Electronic Search Strategy

PubMed (searched through May 2025)

("angiotensin II"[MeSH Terms] OR "angiotensin II"[Title/Abstract] OR "Ang II"[Title/Abstract]) AND ("vasodilatory shock"[Title/Abstract] OR "refractory shock"[Title/Abstract] OR "catecholamine-resistant shock"[Title/Abstract] OR "vasoplegic shock"[Title/Abstract] OR "vasoplegia"[Title/Abstract]) AND ("randomized controlled trial"[Publication Type] OR "RCT"[Title/Abstract] OR "clinical trial"[Title/Abstract]) AND ("mean arterial pressure"[Title/Abstract] OR "MAP"[Title/Abstract] OR "norepinephrine requirement"[Title/Abstract] OR "renal replacement therapy"[Title/Abstract] OR "adverse events"[Title/Abstract])

Scopus (searched through May 2025)

TITLE-ABS-KEY ("angiotensin II" OR "Ang II") AND TITLE-ABS-KEY ("vasodilatory shock" OR "refractory shock" OR "catecholamine-resistant shock" OR "vasoplegic shock" OR "vasoplegia") AND TITLE-ABS-KEY ("randomized controlled trial" OR "RCT" OR "clinical trial") AND TITLE-ABS-KEY ("mean arterial pressure" OR "norepinephrine requirement" OR "renal replacement therapy" OR "adverse events")

Web of Science (searched through May 2025)

TS=("angiotensin II" OR "Ang II") AND TS=("vasodilatory shock" OR "refractory shock" OR "catecholamine-resistant shock" OR "vasoplegia" OR "vasoplegic shock") AND TS=("randomized controlled trial" OR "RCT" OR "clinical trial") AND TS=("mean arterial pressure" OR "norepinephrine" OR "renal replacement therapy" OR "adverse events")
